# Impact of Preoperative Waiting Time on Intraoperative Blood Transfusion in Older Patients with Hip Fracture: A Retrospective Cohort Study

**DOI:** 10.3390/jcm15166193

**Published:** 2026-08-10

**Authors:** Neng-Jun Wang, Wei-Song Zhang, Lin Liu, Bin-Fei Zhang

**Affiliations:** Department of Joint Surgery, Honghui Hospital, Xi’an Jiaotong University, Xi’an 710054, China

**Keywords:** geriatric hip fracture, preoperative waiting time, intraoperative blood transfusion, retrospective cohort study, perioperative management

## Abstract

**Objective:** To explore the associations of preoperative waiting time with the incidence of intraoperative blood transfusion and transfusion volume among older patients with hip fracture, to inform perioperative blood management and optimize surgical timing. **Methods:** Older patients aged ≥65 years who underwent hip fracture surgery at a tertiary trauma center in Northwest China between 1 January 2015 and 30 September 2019 were included. The primary exposure variable was preoperative waiting time. The outcome indicators included intraoperative transfusion status and total transfusion volume. Logistic regression was used for binary transfusion outcomes, and general linear regression was applied for continuous transfusion volume. Multivariate regression models were established to adjust for confounders after univariate screening, and stratified analyses were performed to explore population heterogeneity. **Results:** A total of 2836 eligible patients were included in this study. The mean age of all participants was 79.52 ± 6.68 years, including 2100 cases of intertrochanteric fracture and 736 cases of femoral neck fracture. Intraoperative blood transfusion was administered in 1415 patients (49.9%). Fully adjusted analyses revealed that each one-day increase in preoperative waiting time was associated with a 6% higher odds of intraoperative transfusion (odds ratio [OR] = 1.06, 95% confidence interval [CI]: 1.02–1.10, *p* = 0.0007) and a 0.03-unit increase in transfusion volume (β = 0.03, 95%CI: 0.02–0.05, *p* < 0.0001). However, no independent association was observed for massive transfusion. The associations were generally consistent across strata despite apparent numerical differences (*p* for interaction tests > 0.05). **Conclusions:** Among older patients undergoing hip fracture surgery, longer waiting time was associated with higher transfusion risk. These findings highlight the critical importance of optimizing perioperative pathways to minimize surgical delays. Because residual confounding and inability to distinguish medical from administrative delay remain important limitations, these findings should be interpreted as an association rather than proof that surgical delay causes increased transfusion requirements.

## 1. Introduction

Hip fracture is a traumatic injury associated with substantial disability and mortality among older patients [1,2]. Owing to diminished physiological reserve and high prevalence of multiple chronic comorbidities in older adults, traumatic stress and prolonged bed rest following fracture rapidly deteriorate systemic conditions and markedly increase the risks of complications, including pulmonary infection, deep venous thrombosis, and pressure injury [3,4,5,6]. Early surgical fixation or hip arthroplasty serves as the cornerstone of management for geriatric hip fractures. Domestic and international clinical guidelines recommend that eligible patients with acceptable physical status receive definitive surgery within 24–48 h of admission to facilitate early mobilization and enhanced postoperative recovery [7,8,9].

Previous studies on the timing of surgical treatment for hip fractures predominantly focus on all-cause mortality, postoperative complications, hospital stay, and long-term physical function [10,11,12,13,14,15,16,17,18], whereas the quantitative association between preoperative waiting duration and intraoperative blood transfusion remains insufficiently investigated. Preoperative anemia [19,20,21], persistent hidden blood loss after fracture [22,23], and receiving anticoagulant medication [24,25,26,27] are critical risk factors triggering perioperative allogeneic transfusion. Transfusion is often necessary to maintain intraoperative circulatory stability in patients with severe anemia. However, allogeneic blood is a limited resource and may pose risks for older patients. Furthermore, blood transfusion in older patients raises the risk of diverse transfusion-related adverse reactions [28] and increases hospitalization expenses. From the perspective of blood conservation and perioperative safety, exploring the clinical impacts of preoperative waiting using transfusion-related endpoints can supplement evidence supporting early, timely surgical intervention.

Multiple mechanisms may underlie the independent association between preoperative waiting time and transfusion risk. First, a hip fracture triggers persistent hidden blood and an expanding hematoma at the fracture site and adjacent soft tissues [29,30], prolonged waiting time is associated with persistent hidden blood loss into the tissue spaces surrounding the fracture, leading to a cumulative decrease in hemoglobin levels. Second, anticoagulant therapy administered during the waiting period may further exacerbate anemia [31,32]. Third, trauma and subsequent immobilization trigger a systemic inflammatory response, characterized by elevated cytokines (e.g., IL-6, TNF-α), which can inhibit bone marrow hematopoietic function and erythropoiesis [33,34]. Finally, extended bed rest contributes to physiological deconditioning and reduced physiological reserve, potentially increasing the risk of perioperative complications and bleeding [35]. Thus, we hypothesized that prolonged preoperative waiting was associated with the risk of intraoperative transfusion for older patients with hip fractures.

Accordingly, based on a large single-center retrospective cohort, intraoperative transfusion was selected as the primary endpoint. This choice was made for several reasons. First, it represents a discrete and clearly documented event directly linked to the surgical procedure, minimizing the influence of heterogeneous postoperative management protocols. Second, focusing on intraoperative transfusion provides a more direct measure of the combined effects of preoperative anemia and acute intraoperative blood loss, which are the primary drivers of the need for transfusion during surgery. In contrast, postoperative transfusion is more susceptible to variable clinical practices regarding transfusion triggers and perioperative complications. Therefore, intraoperative transfusion serves as a more consistent and valid measure to evaluate the association of preoperative waiting time with acute surgical blood requirements.

## 2. Materials and Methods

### 2.1. Study Design and Institutional Review Board Approval

This retrospective cohort study enrolled older patients hospitalized for hip fracture at Honghui Hospital, Xi’an Jiaotong University, between 1 January 2015 and 30 September 2019. The study protocol was approved by the Institutional Review Board of Honghui Hospital (approval No. 202201009) and registered at the Chinese Clinical Trial Registry (registration No. ChiCTR2200057323). All study procedures were performed in accordance with the Declaration of Helsinki and relevant ethical regulations. Owing to the retrospective nature of this investigation, the ethics committee waived the requirement for written informed consent. The present study was reported following the Strengthening the Reporting of Observational Studies in Epidemiology (STROBE) guidelines [36,37].

### 2.2. Setting

This study was conducted at the Department of Orthopedic Trauma, Honghui Hospital, Xi’an Jiaotong University. The department established a multidisciplinary team consisting of orthopedics, anesthesiology, geriatrics, rehabilitation, and ultrasonography specialists before 2015. Throughout the study period (2015–2019), this team continuously developed and refined a standardized hip fracture care system, enabling progressively standardized whole-course management including emergency admission, preoperative assessment, surgical intervention, perioperative care and postoperative follow-up for elderly hip fracture patients. The core clinical pathways and transfusion protocols, however, remained consistent during the study period.

### 2.3. Participants

Inclusion criteria: (1) Age ≥ 65 years old; (2) Diagnosed with femoral neck fracture or intertrochanteric fracture confirmed by X-ray, computed tomography (CT) or magnetic resonance imaging (MRI), irrespective of fracture displacement or comminution severity; (3) Hip fracture without an associated open wound; (4) Accompanied by mild non-life-threatening injuries at other anatomical sites was permitted; (5) Received definitive surgical treatment during hospitalization; (6) Complete inpatient clinical and surgical documentation available. Exclusion criteria: (1) Severe multiple trauma involving other body regions; (2) Critically ill patients with high-energy polytrauma induced by motor vehicle collision or high-level fall; (3) Requiring preoperative transfusion; (4) Missing admission or surgical timing data that precluded accurate calculation of preoperative waiting duration. Two trained orthopedic physicians independently reviewed all inpatient records from 1 January 2015 to 30 September 2019 at the Department of Orthopedic Trauma, Honghui Hospital, to screen eligible participants according to predefined criteria. A third senior attending physician adjudicated any controversial cases to guarantee accuracy and consistency of subject enrollment.

### 2.4. Variables

The primary exposure was preoperative waiting time (days). Study outcomes consisted of three indicators: intraoperative transfusion status (yes/no), total intraoperative transfusion volume, and massive transfusion (≥4 units). Transfusion was defined exclusively as the transfusion of red blood cell suspension in additive solution. Collected covariables included age, sex, injury mechanism, fracture classification, age-adjusted Charlson Comorbidity Index (aCCI), hypertension, diabetes mellitus, coronary heart disease (CHD), arrhythmia, associated injuries, time from injury to admission, admission hemoglobin, hematocrit, operative time, intraoperative blood loss, total intraoperative fluid infusion, and stay in hospital. The primary study endpoints were the incidence and volume of intraoperative allogeneic blood transfusions. Based on age and personal health factors, in-hospital management of hip fractures generally falls into three surgical categories: closed/open reduction internal fixation (CRIF/ORIF), hemiarthroplasty (HA), or total hip arthroplasty (THA).

During the study period, our institutional transfusion practice was guided by a general protocol recommending allogeneic red blood cell transfusion for patients with hemoglobin levels below 90 g/L, a threshold consistent with the restrictive transfusion strategies advocated during that era. However, the final decision to transfuse was at the discretion of the attending anesthesiologist and surgeon. This decision was individualized based on the patient’s hemodynamic stability, cardiovascular and pulmonary reserve, comorbidities, ongoing blood loss, and the specific surgical context. While a formal, mandatory protocol was not enforced, this practice remained generally consistent throughout the study period. Complete blood counts were rechecked after each transfusion to evaluate therapeutic response. Complete blood count was rechecked after each transfusion to evaluate therapeutic response, defined as an appropriate rise in hemoglobin/hematocrit levels. Clinical response was also assessed, including improvement in tachycardia, hypotension, and oxygen saturation.

### 2.5. Study Size

As a real-world retrospective observational cohort study designed to explore associations between preoperative waiting duration and transfusion outcomes, identify potential time thresholds and relevant influencing factors, and inform formal pre-study sample size calculations for interventional trials, a formal pre-study sample size calculation was not required. The enrolled sample size was adequate to accommodate multiple statistical analyses and ensure robust, representative, and reliable statistical inferences. Based on the widely accepted 10 events per variable (10 EPV) principle [38] for regression modeling, approximately 25 covariables were incorporated into the regression models; the transfusion rate was about 50%, requiring a minimum of 500 patients with intraoperative blood transfusion to meet statistical reliability requirements, which were fully met in the present cohort.

### 2.6. Quantitative Variables

Continuous variables were categorized into normally distributed and skewed variables based on their distribution characteristics after rigorous data quality control. (1) Normally distributed variables included age, operative duration, and length of hospital stay. Normality was verified via the Shapiro–Wilk test, and data were expressed as mean ± standard deviation. (2) Skewed variables contained time to admission, preoperative waiting time, intraoperative blood loss, intraoperative fluid infusion volume and age-adjusted Charlson Comorbidity Index (aCCI). The aCCI was scored according to chronic disease categories and severity, with higher scores indicating a heavier comorbidity burden. These variables failed normality testing and were presented as median (minimum, maximum). Outlier detection for all continuous variables was performed using box plots after data collection. Any identified outliers were further verified against the original medical records to ensure the authenticity and reliability of the quantitative data.

### 2.7. Statistical Methods

Descriptive statistics included means (SD) and medians (Q1, Q3) for continuous variables, and frequencies (percentages) for categorical variables. Inter-subgroup differences within groups were assessed using χ^2^ tests, one-way ANOVA, or the Kruskal–Wallis H test. Candidate variables for the multivariable models were selected based on a priori clinical importance and established knowledge from the literature on determinants of blood transfusion in hip fracture surgery. To manage the large number of variables, a preliminary univariate screening (*p* < 0.1) was employed as a supplementary guide to identify potential predictors. Ultimately, the final set of variables included in the models was determined through a combination of clinical relevance, literature evidence, and a causal framework. These baseline confounders in Adjusted Model I were fracture classification, aCCI, hypertension, associated injuries, treatment strategy, and hemoglobin. Intraoperative variables (operative duration, blood loss, and fluid infusion) were excluded from this primary model as they may lie on the causal pathway between waiting time and transfusion. Adjusted Model I is intended to estimate the overall effect and is considered our primary confirmatory analysis. A secondary adjusted model (Adjusted Model II) was then constructed by additionally including intraoperative variables (operative duration, blood loss, and fluid infusion). We explicitly acknowledge that these intraoperative factors may lie on the causal pathway between waiting time and transfusion. Therefore, Model II is considered exploratory and was used to investigate potential mediation, rather than as a confirmatory analysis of the total effect. We also note the risk of over-adjustment bias in this model. Given their strong correlation, hemoglobin was retained in the final model while hematocrit was excluded for parsimony. The performance of the final logistic regression model for the binary transfusion outcome was assessed using the area under the receiver operating characteristic curve (AUC) for discrimination and the Hosmer-Lemeshow test for calibration.

For the massive transfusion outcome (≥4 units), which occurred in only 169 patients (6.0% of the total cohort and 11.9% of the transfused cohort), we used penalized (Firth) logistic regression to minimize small-sample bias. To maintain model stability and prevent overfitting, we limited the number of covariates in the Firth regression model. This restricted model included the following key covariates: fracture classification, aCCI, hypertension, associated injuries, treatment strategy, and hemoglobin (6 covariates plus the exposure variable). This resulted in an EPV ratio of approximately 24.1 (169 events/7 predictors), which exceeds the recommended minimum of 10 events per variable for reliable logistic regression, supporting the stability of our estimates. The use of Firth’s penalized likelihood method further reduces the risk of bias in this small-sample context.

Multicollinearity was assessed using the Variance Inflation Factor (VIF); all variables in the final models had VIF values < 5, indicating no significant multicollinearity.

The primary association was assessed with a general linear model and logistic regression. Analyses were conducted in R and EmpowerStats, and β and odds ratios (ORs) with 95% confidence intervals (CIs) were reported. A two-sided *p* < 0.05 indicated statistical significance.

## 3. Results

### 3.1. Patient Characteristics

**Figure 1:**
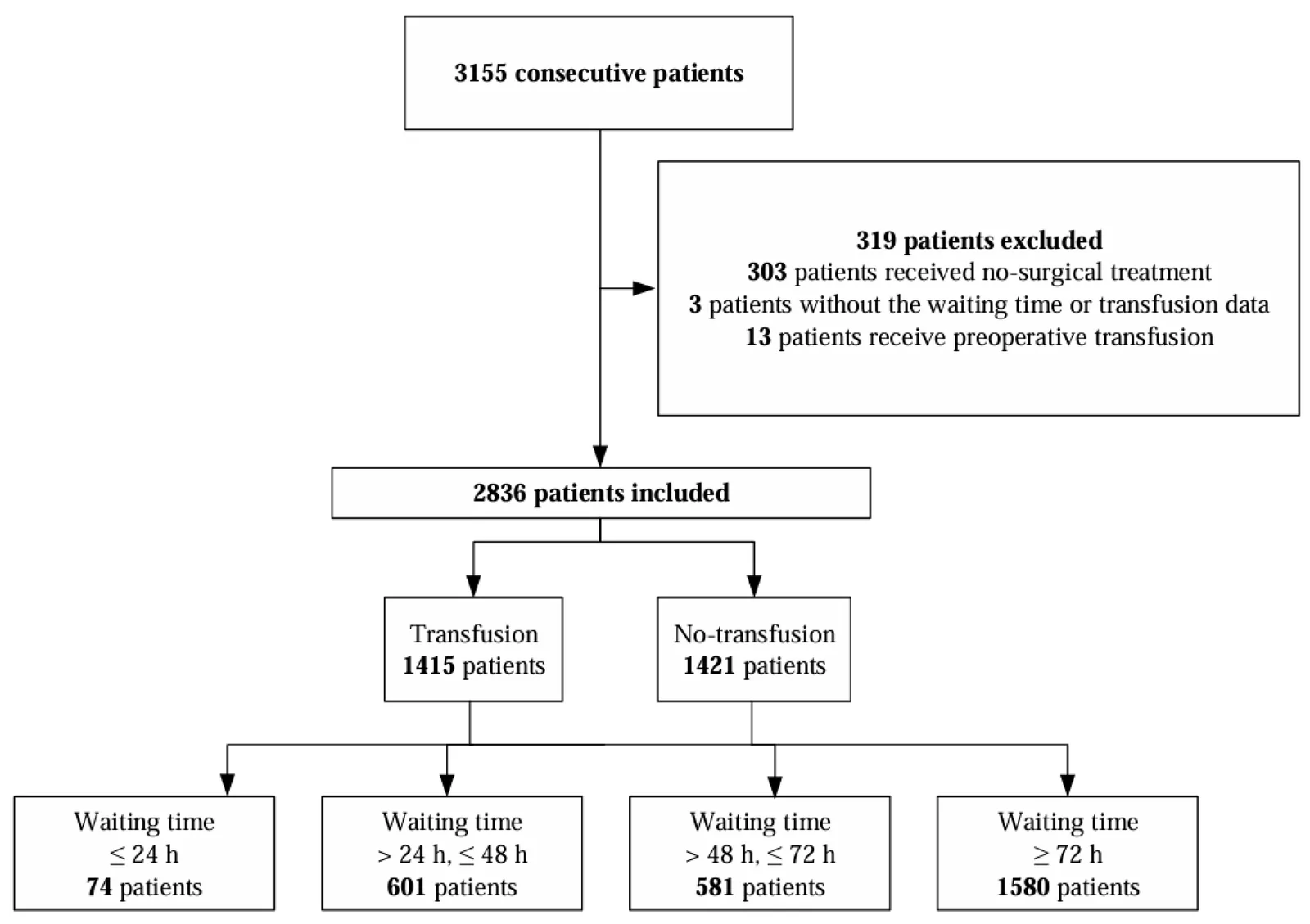
The screening and exclusion process in this study.

A total of 3155 patients were initially screened. After applying exclusion criteria (319 patients: 303 with non-surgical treatment, 13 with preoperative transfusion, and 3 without data on waiting time or transfusion), 2836 eligible patients were included in the final analysis (Figure 1). The final dataset had no missing data for the key exposure, outcome, and adjusted variables. There were 894 males (31.5%) and 1942 females (68.5%), with a mean age of 79.52 ± 6.68 years. There were 2100 cases (74.1%) of intertrochanteric fracture and 736 cases (25.9%) of femoral neck fracture. Intraoperative allogeneic blood transfusion was administered in 1415 patients (49.9%), and detailed baseline characteristics are summarized in Table 1.

Compared with patients without transfusion, transfused patients were significantly older (80.47 ± 6.60 years vs. 78.57 ± 6.62 years, *p* < 0.001), had a higher proportion of intertrochanteric fracture (87.0% vs. 61.2%, *p* < 0.001), elevated aCCI scores (*p* < 0.001), higher rates of concomitant injuries (*p* < 0.001), prolonged preoperative waiting time (4 (2–5) days vs. 4 (3–5) days, *p* = 0.002) and longer hospital stay (*p* < 0.001). Additionally, the transfusion group had lower preoperative hemoglobin (*p* < 0.001) and hematocrit levels (*p* < 0.001), longer operative duration (*p* < 0.001), and greater intraoperative blood loss (*p* < 0.001). The mean transfusion volume among recipients was 2.25 ± 0.73 units, and massive transfusion (≥4 units) occurred in 169 patients.

Baseline comparisons revealed that patients receiving transfusions had prolonged preoperative waiting times alongside more complicated perioperative profiles. A substantial standardized mean difference was observed for fracture subtype, indicating prominent clustering of intertrochanteric fractures in the transfusion cohort. Marked between-group differences in hemoglobin and hematocrit suggested that preoperative anemia and insufficient hematologic reserve were important predisposing factors for transfusion requirements. Significant differences in operative duration and intraoperative blood loss further demonstrated that surgical complexity and actual hemorrhagic burden remained central determinants guiding intraoperative transfusion decisions.

### 3.2. Univariate Analysis of the Association Between Variables and Blood Transfusion

Univariate regression analyses demonstrated that preoperative waiting time was significantly associated with all three transfusion-related outcomes. Each additional day of preoperative waiting was correlated with a 5% increase in the risk of intraoperative transfusion (OR = 1.05, 95%CI: 1.02–1.09, *p* = 0.0006), a 0.04-unit rise in transfusion volume (β = 0.04, 95%CI: 0.02–0.05, *p* < 0.0001), and a 7% higher risk of massive transfusion (≥4 units) (OR = 1.07, 95%CI: 1.02–1.13, *p* = 0.0038). Multiple other variables, including age, fracture classification, aCCI, hypertension, concomitant injuries, gastritis, preoperative hemoglobin, hematocrit, surgical modality, operative duration, intraoperative blood loss, intraoperative fluid infusion, and length of hospital stay, also showed varying degrees of correlation with transfusion outcomes, as shown in Table 2.

Patients with femoral neck fracture exhibited lower transfusion risk and smaller transfusion volume compared with those with intertrochanteric fracture. Additionally, hemiarthroplasty was associated with a lower transfusion risk relative to internal fixation. Higher preoperative hemoglobin and hematocrit levels were protective against all three transfusion outcomes, whereas prolonged operative duration and increased intraoperative blood loss were linked to elevated transfusion risk.

We used clinical relevance, previously reported factors, a directed acyclic graph, and variables meeting a *p* < 0.1 threshold in univariate analysis to identify confounding factors for multivariable modeling. Finally, the following variables were included in the multivariable regression models: fracture classification, aCCI, hypertension, associated injuries, treatment strategy, and hemoglobin.

### 3.3. Multivariate Analysis of Waiting Time and Blood Transfusion

Multivariate analysis confirmed that longer preoperative waiting time was independently associated with higher intraoperative transfusion risk and volume. Multicollinearity was assessed using VIF, and all variables in the final models had VIF < 5. Fully adjusted analyses revealed that each one-day increase in preoperative waiting time was associated with a 6% higher risk of intraoperative transfusion (OR = 1.06, 95%CI: 1.02–1.09, *p* = 0.0007) and a 0.03-unit increase in transfusion volume (β = 0.03, 95%CI: 0.02–0.05, *p* < 0.0001), showing in Table 3. The primary logistic regression model (Adjusted Model I) for intraoperative transfusion demonstrated good discrimination, with an AUC of 0.80 (95%CI: 0.78–0.82), and good calibration, as indicated by a non-significant Hosmer-Lemeshow test (χ = 8.92, *p* = 0.35). A secondary adjusted model II including intraoperative variables (operation time, blood loss, infusion) was performed as a sensitivity analysis to explore potential mediation effects. The result showed that preoperative waiting time was associated with intraoperative transfusion and transfusion volume.

Regarding the exploration of the association between ATO and massive transfusion (≥4 units). Massive transfusion occurred in only 169 patients. Even though significant associations were observed in the non-adjusted model and adjusted model I, each one-day increase in preoperative waiting time was associated with a 7% higher risk of intraoperative massive transfusion, but not in adjusted model II (OR = 1.06, 95%CI: 0.98–1.13, *p* = 0.1476).

### 3.4. Sensitivity Analysis

To address the zero-inflated and discrete nature of the transfusion volume data, we applied a generalized linear model with a Poisson distribution and robust standard errors, restricted to the 1415 patients who received intraoperative transfusion. The result showed that each additional day of preoperative waiting was associated with a 0.03-unit increase in transfusion volume (β = 0.03, 95% CI: 0.01–0.04, *p* = 0.0003) after adjustment for baseline confounders. This estimate was nearly identical to that from the primary linear regression model (β = 0.03, 95% CI: 0.01–0.04, *p* = 0.0002), confirming the robustness of our findings.

We assessed potential non-linear association using general additive model analysis. We found no significant departure from linearity (*p* for non-linearity > 0.05 for all outcomes), supporting the use of linear models in the primary analysis (Supplementary Table S1).

### 3.5. Stratified Analysis

Stratified analyses showed that prolonged preoperative waiting time was significantly correlated with a higher intraoperative transfusion rate and larger transfusion volume in both patients with intertrochanteric fracture and femoral neck fracture. The associations were statistically significant in patients aged 65–75 years and 75–85 years, but not in those aged 85 years or older. For male patients, preoperative waiting time was significantly associated with both transfusion occurrence and transfusion volume. Among female patients, waiting time was correlated with transfusion incidence, while no significant association was found with transfusion volume. When stratified by time from injury to admission, positive correlations between preoperative waiting time and the two transfusion outcomes were observed in both subgroups (≤24 h and >24 h). Stratification by aCCI revealed stable associations in patients with aCCI ≤ 4, whereas no significant relationships were detected in those with aCCI > 4 (Table 4).

For the outcome of massive transfusion (transfusion volume ≥ 4 units), a significant association between preoperative waiting time and massive transfusion was only identified in patients aged 65–75 years and those with aCCI ≤ 4.

However, the interaction tests revealed no statistically significant subgroup differences (all *p* for interaction > 0.05), suggesting that the associations were generally consistent across strata despite apparent numerical differences.

## 4. Discussion

This retrospective cohort study demonstrated that each additional day of waiting time was associated with a 6% increase in the odds of intraoperative blood transfusion and a 0.03-unit increase in transfusion volume. However, no independent association was observed for massive transfusion. The associations were generally consistent across strata despite apparent numerical differences. While the association between waiting time and transfusion volume was statistically significant, the effect size was small (β = 0.03 units per day). An absolute increase of 0.09 units for a 3-day delay is unlikely to be clinically significant at the individual patient level. However, the clinical and public health significance of this finding should be evaluated in the context of the broader population. For a high-volume center managing hundreds or thousands of such patients annually, a small per-patient effect can translate into a substantial cumulative demand on blood product inventories. Furthermore, the primary clinical outcome remains the binary transfusion decision (a 6% increase in odds per day), which may be more directly relevant at the bedside. Our findings support prioritizing timely surgery as a component of a comprehensive, system-level patient blood management strategy, rather than as a means to achieve a large, individually measurable reduction in transfusion volume.

Current studies on surgical timing for hip fractures mainly focus on mortality, postoperative complications, and length of hospital stay [10,11,12,13,14,15,16,17,18], while research taking blood transfusion as the primary outcome remains scarce. The existing literature generally supports the notion that early surgery is associated with overall complication risk, yet evidence on transfusion as a specific endpoint remains inconsistent. Consistent with our findings, several studies have reported that prolonged preoperative waiting is linked to aggravated postoperative anemia and a higher transfusion rate, particularly in patients with intertrochanteric fractures [27,39,40].

Leif Mattisson et al. demonstrated that surgical delay beyond 24 h was significantly associated with a higher rate of preoperative transfusion, with a relative risk of 3.0 [27]. Su et al. reported that patients undergoing surgery within 48 h had a markedly lower preoperative transfusion rate and smaller perioperative blood loss than those undergoing surgery after 48 h (15.63% vs. 26.53%; 80.21 ± 196.63 mL vs. 144.90 ± 253.52 mL) [40]. Nevertheless, these studies only compared differences between two groups without quantifying effect sizes, and few adjusted for key confounders, including fracture type, preoperative hemoglobin level, surgical modality, and intraoperative blood loss. Our findings on the association between prolonged waiting and transfusion risk in a large real-world cohort may help generate hypotheses for future prospective trials with transfusion as a primary endpoint.

The present study is distinguished by evaluating three transfusion-related outcomes simultaneously: transfusion status, total transfusion volume, and massive transfusion (≥4 units). Transfusion status indicates whether the clinical threshold for transfusion is reached. Transfusion volume reflects blood resource consumption and the continuous burden of anemia and blood loss. A comprehensive analysis of these outcomes provides a full understanding of the association between preoperative waiting time and perioperative blood management.

Furthermore, no significant association was observed among patients aged 85 years or older or those with a high comorbidity burden. The transfusion rate reached 58.49% in patients aged 85 years or older, which was higher than that in younger patients, and a similar trend was observed in the subgroup with an aCCI > 4. A plausible explanation is that transfusion decisions for these populations are influenced by multiple confounding factors, such as preoperative optimization strategies and clinicians’ judgments about tolerance to intraoperative blood loss in the oldest-old, thereby attenuating the independent effect of waiting time. Overall, our study employed rigorous methods to control for confounders, revealing the independent associations between preoperative waiting time and transfusion outcomes at different levels. It also fills the evidence gap concerning intraoperative transfusion volume in this field.

Multiple mechanisms may underlie the independent association between preoperative waiting time and transfusion risk. First, a hip fracture triggers persistent occult bleeding and expanding hematoma at the fracture site and adjacent soft tissues [41]. Combined with traumatic stress and hemodilution caused by liberal intravenous fluid administration [42], anemia progressively deteriorates as waiting time increases. Surgical delay perpetuates these processes and, consequently, increases the demand for intraoperative blood transfusion. Second, anticoagulant therapy administered during the waiting period [24,25,26,27] and inadequate nutritional support may further exacerbate anemia. In addition, prolonged waiting time can sustain systemic inflammatory stress, inhibit bone marrow hematopoietic function [43], and further compromise patients’ physiological reserve [44,45].

However, preoperative waiting time may also reflect underlying disease complexity [46,47,48]. Patients with severe cardiopulmonary diseases, infection, or coagulation disorders often require prolonged preoperative assessment and medical optimization, and these conditions are inherently linked to an elevated transfusion risk. A key methodological consideration in our analysis was the selection of variables for multivariate adjustment. We employed a two-model strategy to best elucidate the relationship between preoperative waiting time and transfusion. Our primary analysis (Adjusted Model I) focused on estimating the total effect of waiting time by adjusting for baseline confounders (e.g., age, fracture type, comorbidity burden, preoperative hemoglobin) that precede the surgery. We intentionally excluded intraoperative variables such as operative duration and intraoperative blood loss from this primary model. This is because these factors are likely to be on the causal pathway; that is, a longer waiting time may lead to a more complex surgery with greater blood loss, which in turn necessitates a transfusion. Adjusting for such mediators in the primary model could obscure the total effect of the waiting time itself. To address this and test the robustness of our findings, we performed a secondary analysis (Adjusted Model II) that included these intraoperative variables. The persistence of a significant association in this more fully adjusted model suggests that the effect of preoperative waiting time on transfusion risk is not entirely mediated by operative duration or measured blood loss, and may involve other mechanisms. This two-model approach provides a more nuanced understanding of the association. However, residual confounding from unmeasured factors could not be ruled out entirely.

This study indicates that reducing avoidable preoperative waiting time may help decrease intraoperative transfusion exposure among older patients with hip fractures, conserve limited allogeneic blood resources, and lower the risk of transfusion-related adverse reactions. For patients who cannot undergo immediate surgery due to medical conditions, the waiting period should not be regarded as a passive observation phase but transformed into a window for active intervention. Orthopedic surgeons should dynamically monitor hemoglobin levels, assess coagulation function, standardize anemia management, avoid excessive fluid overload, and formulate advanced plans for blood preparation and transfusion [49,50,51]. Furthermore, medical institutions are recommended to establish a multidisciplinary fast-track pathway for older hip fracture care [51,52], which integrates surgical timing, rational use of blood products, anemia screening and transfusion strategies into a standardized management protocol. In future hospital quality improvement initiatives, transfusion rate and transfusion volume can be adopted as key indicators to evaluate the overall performance of perioperative management.

This study had a large sample size and enrolled patients with the two most common types of hip fractures: femoral neck fractures and intertrochanteric fractures. Three transfusion-related outcomes, including transfusion status, transfusion volume, and massive transfusion, were analyzed simultaneously, which is more consistent with real clinical practice. We adjusted for key confounding variables in the multivariate models, covering demographic characteristics, fracture classification, comorbidity burden, preoperative hematological parameters, surgical modality, operative duration, intraoperative blood loss and fluid infusion. Stratified analyses were also conducted to assess the consistency of the associations across different subgroups.

Nevertheless, several limitations should be noted. First, the retrospective design made it impossible to confirm causal relationships and restricted the generalizability of the results. Second, transfusion decisions were affected by clinicians’ practice patterns and changing transfusion criteria over time, and uniform transfusion indications were not recorded in this study. Third, several potential confounders were not incorporated in the study. These include but are not limited to ASA physical status classification, frailty scores, detailed medication profiles (especially anticoagulant/antiplatelet therapies), specific laboratory values such as INR and renal function markers, the use of tranexamic acid, and detailed perioperative anemia management protocols. Furthermore, unmeasured factors such as surgeon and anesthesiologist practice preferences could have influenced both the decision to delay surgery and the threshold for transfusion. The retrospective nature of our study precluded reliable collection of these data, and their omission represents a significant source of potential residual confounding. The absence of this detailed information, particularly on the specific reasons for surgical delays, means our findings should be interpreted as an association rather than definitive evidence. Fourth, this study was limited by the inability to definitively distinguish between medically indicated and administrative delays. In our electronic medical record system, the specific reason for each surgical delay was not reliably coded, preventing a precise classification. This is a significant limitation because medically unstable patients may experience longer waits precisely because they require optimization for conditions that themselves increase transfusion risk, potentially biasing our estimates. Therefore, our findings should be interpreted as an association rather than definitive evidence.

## 5. Conclusions

Among older patients undergoing hip fracture surgery, longer waiting time was associated with higher transfusion risk. These findings highlight the critical importance of optimizing perioperative pathways to minimize surgical delays. Because residual confounding and inability to distinguish medical from administrative delay remain important limitations, these findings should be interpreted as an association rather than proof that surgical delay causes increased transfusion requirements.

## Figures and Tables

**Table 1:** Baseline demographic and clinical characteristics of the study population, stratified by intraoperative transfusion status.

Transfusion	No	Yes	Standardized Diff.	*p*-Value ^†^	*p*-Value *
N	1421	1415			
Age (year)	78.57 ± 6.62	80.47 ± 6.60	0.29 (0.21, 0.36)	<0.001	<0.001
Sex			0.04 (−0.03, 0.11)	0.291	-
Male	461 (32.44%)	433 (30.60%)			
Female	960 (67.56%)	982 (69.40%)			
Injury mechanism			0.02 (−0.06, 0.09)	0.913	-
Falling	1376 (96.83%)	1374 (97.10%)			
Accident	37 (2.60%)	34 (2.40%)			
Other	8 (0.56%)	7 (0.49%)			
Fracture classification			0.62 (0.54, 0.69)	<0.001	-
Intertrochanteric fracture	869 (61.15%)	1231 (87.00%)			
Femoral neck fracture	552 (38.85%)	184 (13.00%)			
aCCI	4.08 ± 1.08	4.27 ± 1.02	0.18 (0.10, 0.25)	<0.001	<0.001
Hypertension	746 (52.50%)	643 (45.44%)	0.14 (0.07, 0.22)	<0.001	-
Diabetes	288 (20.27%)	254 (17.95%)	0.06 (−0.01, 0.13)	0.117	-
CHD	719 (50.60%)	742 (52.44%)	0.04 (−0.04, 0.11)	0.327	-
Arrhythmia	445 (31.32%)	482 (34.06%)	0.06 (−0.02, 0.13)	0.119	-
Associated injuries	75 (5.28%)	124 (8.76%)	0.14 (0.06, 0.21)	<0.001	-
Time to admission (h) ^#^	10 (4–72)	10 (4–72)	0.00 (−0.07, 0.07)	0.971	0.646
Preoperative waiting time (d) ^#^	4 (2–5)	4 (3–5)	0.13 (0.06, 0.21)	<0.001	0.002
≤24 h	44 (3.10%)	30 (2.12%)			
>24 h, ≤48 h	327 (23.01%)	274 (19.36%)			
>48 h, ≤72 h	283 (19.92%)	298 (21.06%)			
≥72 h	767 (53.97%)	813 (57.46%)			
Stay in hospital (d)	8.44 ± 3.40	9.06 ± 3.45	0.18 (0.11, 0.25)	<0.001	<0.001
Hemoglobin (g/L)	117.65 ± 17.69	104.75 ± 18.36	0.72 (0.64, 0.79)	<0.001	<0.001
Hematocrit (%)	35.60 ± 5.12	31.81 ± 5.35	0.72 (0.65, 0.80)	<0.001	<0.001
Coagulation parameters	±				
Prothrombin time (s)	12.74 ± 1.36	12.94 ± 1.05	0.16 (0.09, 0.24)	<0.001	<0.001
Activated partial thromboplastin time (s)	30.04 ± 4.86	30.87 ± 4.83	0.17 (0.10, 0.25)	<0.001	<0.001
Platelet count (10^9^/L)	183.65 ± 72.32	183.11 ± 80.99	0.01 (−0.07, 0.08)	0.852	0.152
Treatment strategy			0.58 (0.50, 0.65)	<0.001	-
CRIF/ORIF	873 (61.44%)	1203 (85.02%)			
HA	529 (37.23%)	187 (13.22%)			
THA	19 (1.34%)	25 (1.77%)			
Operation time (mins) ^#^	80 (70–100)	90 (70–120)	0.39 (0.32, 0.47)	<0.001	<0.001
Blood loss (mL) ^#^	150 (150–200)	250 (150–400)	0.79 (0.71, 0.86)	<0.001	<0.001
Infusion (mL) ^#^	1600 (1300–1600)	1600 (1100–1600)	0.12 (0.04, 0.19)	0.002	<0.001
Transfusion (U)	0.00 ± 0.00	2.25 ± 0.73	4.39 (4.26, 4.53)	<0.001	<0.001
Transfusion (U)			0.52 (0.45, 0.60)	<0.001	-
<4	1421 (100.00%)	1246 (88.06%)			
≥4	0 (0.00%)	169 (11.94%)			

Data are presented as mean ± SD for normally distributed continuous variables, ^#^ median (Q1, Q3) for skewed continuous variables, and n (%) for categorical variables. ^†^ Parameter test * Nonparametric tests. For continuous variables, we used the Kruskal–Wallis rank-sum test; for count variables with a theoretical count < 10, we used Fisher’s exact test. Abbreviation: CHD, coronary heart disease; HA, hemiarthroplasty; ORIF, open reduction and internal fixation; THA, total hip arthroplasty.

**Table 2:** Univariate analysis of the association between each variable and transfusion outcomes.

	Statistics	Transfusion (No/Yes)	Transfusion Volume	Transfusion Volume ≥ 4 U
Age (y)	79.52 ± 6.68	1.04 (1.03, 1.06) <0.0001	0.02 (0.01, 0.03) <0.0001	1.01 (0.98, 1.03) 0.5113
Sex				
Male	894 (31.52%)	1	0	1
Female	1942 (68.48%)	1.09 (0.93, 1.28) 0.2914	0.02 (−0.08, 0.12) 0.7296	0.85 (0.61, 1.18) 0.3287
Injury mechanism				
Falling	2750 (96.97%)	1	0	1
Accident	71 (2.50%)	0.92 (0.57, 1.47) 0.7298	0.15 (−0.14, 0.44) 0.3191	2.38 (1.16, 4.88) 0.0178
Other	15 (0.53%)	0.88 (0.32, 2.42) 0.7991	0.35 (−0.28, 0.98) 0.2787	2.52 (0.56, 11.28) 0.2256
Fracture classification				
Intertrochanteric fracture	2100 (74.05%)	1	0	1
Femoral neck fracture	736 (25.95%)	0.24 (0.19, 0.28) <0.0001	−0.82 (−0.92, −0.72) <0.0001	0.11 (0.05, 0.25) <0.0001
aCCI	4.17 ± 1.06	1.18 (1.10, 1.27) <0.0001	0.08 (0.04, 0.13) 0.0001	1.02 (0.88, 1.18) 0.7786
Hypertension	1389 (48.98%)	0.75 (0.65, 0.87) 0.0002	−0.16 (−0.26, −0.07) 0.0004	0.86 (0.63, 1.18) 0.3601
Diabetes	542 (19.11%)	0.86 (0.71, 1.04) 0.1168	−0.08 (−0.19, 0.04) 0.1814	0.91 (0.61, 1.36) 0.6430
CHD	1461 (51.52%)	1.08 (0.93, 1.25) 0.3270	0.04 (−0.05, 0.14) 0.3373	1.02 (0.75, 1.40) 0.8817
Arrhythmia	927 (32.69%)	1.13 (0.97, 1.33) 0.1189	0.06 (−0.04, 0.15) 0.2541	0.94 (0.67, 1.31) 0.7048
Associated injuries	199 (7.02%)	1.72 (1.28, 2.32) 0.0003	0.45 (0.27, 0.63) <0.0001	2.36 (1.49, 3.73) 0.0002
Hemoglobin (g/L)	111.21 ± 19.15	0.96 (0.96, 0.97) <0.0001	−0.02 (−0.02, −0.02) <0.0001	0.96 (0.95, 0.97) <0.0001
Hematocrit (%)	33.71 ± 5.57	0.87 (0.86, 0.88) <0.0001	−0.08 (−0.09, −0.07) <0.0001	0.87 (0.85, 0.90) <0.0001
Time to admission (h) ^#^	10 (4–72)	1.00 (1.00, 1.00) 0.9711	−0.00 (−0.00, 0.00) 0.6076	1.00 (1.00, 1.00) 0.2514
Preoperative waiting time (d) ^#^	4 (3–5)	1.05 (1.02, 1.09) 0.0006	0.04 (0.02, 0.05) <0.0001	1.07 (1.02, 1.13) 0.0038
Coagulation parameters				
Prothrombin time (s)	12.84 ± 1.22	1.17 (1.09, 1.26) <0.0001	0.09 (0.05, 0.12) <0.0001	1.12 (1.02, 1.23) 0.0140
Activated partial thromboplastin time (s)	30.45 ± 4.86	1.04 (1.02, 1.05) <0.0001	0.02 (0.01, 0.03) 0.0001	1.01 (0.98, 1.04) 0.6551
Platelet count (10^9^/L)	183.38 ± 76.76	1.00 (1.00, 1.00) 0.8519	−0.00 (−0.00, 0.00) 0.5913	1.00 (1.00, 1.00) 0.4057
Treatment strategy				
CRIF/ORIF	2076 (73.20%)	1	0	1
HA	716 (25.25%)	0.26 (0.21, 0.31) <0.0001	−0.77 (−0.88, −0.67) <0.0001	0.12 (0.06, 0.26) <0.0001
THA	44 (1.55%)	0.95 (0.52, 1.74) 0.8806	−0.00 (−0.36, 0.35) 0.9906	1.21 (0.43, 3.44) 0.7150
Operation time (mins) ^#^	90 (70–110)	1.01 (1.01, 1.01) <0.0001	0.01 (0.01, 0.01) <0.0001	1.02 (1.02, 1.03) <0.0001
Blood loss (mL) ^#^	200 (150–300)	1.01 (1.01, 1.01) <0.0001	0.00 (0.00, 0.00) <0.0001	1.01 (1.01, 1.01) <0.0001
Infusion (mL) ^#^	1600 (1200–1600)	1.00 (1.00, 1.00) 0.0018	0.00 (−0.00, 0.00) 0.4602	1.00 (1.00, 1.00) <0.0001
Stay in hospital (d)	8.75 ± 3.44	1.06 (1.03, 1.08) <0.0001	0.04 (0.03, 0.05) <0.0001	1.09 (1.05, 1.13) <0.0001

Data are presented as OR (95% CI) or β (95% CI) with *p*-values. ^#^ median (Q1, Q3) for skewed continuous variables, and n (%) for categorical variables. Abbreviation: CHD, coronary heart disease; HA, hemiarthroplasty; ORIF, open reduction and internal fixation; THA, total hip arthroplasty.

**Table 3:** Multivariable analysis of the association between preoperative waiting time and transfusion outcomes.

Outcome		Non-Adjusted Model	Adjusted Model I	Adjusted Model II
Transfusion ^†^	No	Ref.	Ref.	Ref.
	Yes	1.05 (1.02, 1.09) 0.0006	1.06 (1.02, 1.09) 0.0007	1.07 (1.03, 1.11) 0.0002
Transfusion Volume *		0.04 (0.02, 0.05) <0.0001	0.03 (0.02, 0.05) <0.0001	0.03 (0.01, 0.04) 0.0002
Transfusion Volume ^†^	<4 U	Ref.	Ref.	Ref.
	≥4 U	1.07 (1.02, 1.13) 0.0038	1.07 (1.01, 1.13) 0.0166	1.05 (0.98, 1.13) 0.1476

Data in the table: * β (95%CI) *p*-value/^†^ OR (95%CI) *p*-value Outcome variate: Transfusion (No/Yes), Transfusion Volume, Transfusion Volume ≥ 4 U Exposure variable: Waiting time (d) Adjusted model I adjusted for fracture classification, aCCI, hypertension, associated injuries, treatment strategy, and hemoglobin. Adjusted model II adjusted for age, fracture classification, aCCI, hypertension, associated injuries, treatment strategy, operation time, blood loss, infusion, hemoglobin, and hematocrit.

**Table 4:** Stratified analysis of the association between preoperative waiting time and transfusion outcomes across subgroups.

Subgroup	No. of Patients	No. of Transfusion (%)	Transfusion (No/Yes)	Transfusion Volume	Transfusion Volume ≥ 4 U	*p* for Interaction *
Fracture classification						
Intertrochanteric fracture	2100	1231 (58.62%)	1.07 (1.03, 1.12) 0.0019	0.03 (0.01, 0.05) 0.0026	1.05 (0.97, 1.13) 0.2466	0.9772
Femoral neck fracture	736	184 (25.00%)	1.09 (1.01, 1.17) 0.0238	0.03 (0.01, 0.05) 0.0146	1.17 (0.99, 1.39) 0.0703	
Age						
65 ≤ age ≤ 75	792	321 (40.53%)	1.08 (1.01, 1.15) 0.0214	0.04 (0.01, 0.06) 0.0027	1.18 (1.07, 1.32) 0.0015	0.2986
75 < age ≤ 85	1514	784 (51.78%)	1.08 (1.02, 1.14) 0.0055	0.03 (0.01, 0.05) 0.0066	1.02 (0.92, 1.13) 0.6857	
85 < age	530	310 (58.49%)	1.05 (0.97, 1.14) 0.2535	0.00 (−0.03, 0.04) 0.8035	0.86 (0.69, 1.08) 0.2081	
Sex						
Male	894	433 (48.43%)	1.10 (1.03, 1.16) 0.0024	0.04 (0.02, 0.06) 0.0010	1.06 (0.97, 1.16) 0.2028	0.2201
Female	1942	982 (50.57%)	1.05 (1.00, 1.11) 0.0405	0.02 (−0.00, 0.04) 0.0690	1.02 (0.92, 1.14) 0.6807	
Time to admission						
≤24 h	1949	971 (49.82%)	1.09 (1.04, 1.15) 0.0010	0.03 (0.01, 0.05) 0.0024	1.04 (0.94, 1.15) 0.4411	0.2180
>24 h	887	444 (50.06%)	1.06 (1.00, 1.12) 0.0462	0.03 (0.00, 0.05) 0.0223	1.07 (0.97, 1.18) 0.2091	
aCCI						
≤4	1876	898 (47.87%)	1.08 (1.03, 1.13) 0.0008	0.03 (0.02, 0.05) 0.0002	1.09 (1.01, 1.18) 0.0255	0.7467
>4	960	517 (53.85%)	1.07 (0.99, 1.15) 0.0688	0.02 (−0.01, 0.04) 0.1715	0.97 (0.85, 1.12) 0.7229	

*p* for interaction was calculated by including the interaction term between the stratification variable and preoperative waiting time in the regression model. Data in the table: β (95%CI) *p*-value/OR (95%CI) *p*-value Outcome variate: Transfusion (No/Yes), Transfusion Volume, Transfusion Volume ≥ 4 U Exposure variable: Waiting time (d). Adjusted variables: age, fracture classification, aCCI; hypertension, associated injuries, treatment strategy, hemoglobin and hematocrit. * *p* for interaction values were calculated by including the interaction term between the stratification variable and preoperative waiting time in the regression model.

## Data Availability

Xi’an Honghui Hospital implemented the data. According to relevant regulations, the data cannot be shared but can be requested from the corresponding author.

## Supplementary Materials

The following supporting information can be downloaded at: https://www.mdpi.com/article/10.3390/jcm15166193/s1, Table S1. The non-linearity association using general additive model analysis.
