# Supplementary material for: Impact of Preoperative Waiting Time on Intraoperative Blood Transfusion in Older Patients with Hip Fracture: A Retrospective Cohort Study
Source: J Clin Med. 2026 Aug 10;15(16):6193. doi: 10.3390/jcm15166193 (PMC13513797; doi:10.3390/jcm15166193)
Supplement: Supplementary file 1 [file jcm-15-06193-s001.zip › jcm-4426963-supplementary.pdf]

Supplementary Table S1. The non-linearity association using general additive model analysis

Outcome	Transfusion (No/Yes)	Transfusion Volume	Transfusion Volume ≥4U
Fitting a model by the standard linear regression	1.06 (1.02, 1.10) 0.0007	0.03 (0.02, 0.05) <0.0001	1.07 (1.01, 1.13) 0.0166
Fitting model by two-piecewise non-linear regression			
Inflection point	7	2	2
< Inflection point	1.04 (0.99, 1.10) 0.1145	0.13 (-0.15, 0.40) 0.3641	2.85 (0.39, 21.09) 0.3043
> Inflection point	1.09 (1.01, 1.17) 0.0242	0.03 (0.02, 0.05) 0.0002	1.06 (1.01, 1.12) 0.0283
P for log-likelihood ratio test	0.435	0.503	0.259
